# Maternal omentin-1 level, quality of life and marital satisfaction in relation to mode of delivery: a prospective cohort study

**DOI:** 10.1186/s12884-020-2825-2

**Published:** 2020-03-02

**Authors:** Simin Parvanehvar, Najmeh Tehranian, Anoshirvan Kazemnejad, Hossein Mozdarani

**Affiliations:** 1 Department of Reproductive Health and Midwifery, Faculty of Medical Sciences, Tarbiat Modares University, Tehran, Iran; 2 Department of Biostatistics, Faculty of Medical Sciences, Tarbiat Modares University, Tehran, Iran; 3 Department of Medical Genetics -Medical Cytogenetic, Faculty of Medical Sciences, Tarbiat Modares University, Tehran, Iran

**Keywords:** Omentin-1, Vaginal delivery, Cesarean section, Quality of life, Marital satisfaction

## Abstract

**Background:**

The purpose of this study was to evaluate the maternal omentin-1 level, quality of life and marital satisfaction of women with cesarean and vaginal delivery.

**Methods:**

This prospective cohort study was conducted on 45 women with elective cesarean delivery and 45 women with vaginal delivery who referred to a public hospital in Tehran, Iran. Maternal omentin-1 level was measured by ELISA kits within 24 h after delivery. The maternal quality of life and marital satisfaction in the third trimester of pregnancy and at 12 weeks postpartum were measured using WHOQOL-BREF and the Kansas marital satisfaction questionnaires, respectively. For making between-groups and within-groups comparison, independent samples t-test, paired samples t-test and chi-square test were applied accordingly.

**Results:**

The level of maternal omentin-1 was reported to be higher in vaginal delivery group compared to the cesarean group (*p* = 0.02). No significant difference was found in the quality of life between the two groups in the third trimester of pregnancy and at 12 weeks postpartum period. However, women in both groups had lower scores in physical dimension at 12 weeks postpartum compared to the third trimester of their pregnancy [mean ± SD in vaginal group = 59.28 ± 15.5 vs. 64.44 ± 15.05, *p* = 0.003 and mean ± SD in cesarean group = 60.07 ± 14.84 vs. 66.50 ± 11.32, *p* <  0.001]. The results of paired samples t-test indicated that women in NVD group had significantly higher psychological wellbeing at 12 weeks postpartum compared to the third trimester of pregnancy [mean ± SD 68.9 ± 16.82 vs. 65.73 ± 16.87, *p* = 0.001]. There was no significant difference in marital satisfaction between the two groups at 12 weeks postpartum (*P* = 0.07). The results of paired samples t-test showed that women in CS group had significantly lower marital satisfaction at 12 weeks postpartum compared to the third trimester of pregnancy [mean SD 18.86 ± 2.04 vs. 19.28 ± 1.79, *p* = 0.01].

**Conclusions:**

Our findings demonstrated that women with NVD had higher omentin-1 level than women with CS. No significant difference was found in quality of life and marital satisfaction between NVD and CS and omentin-1 level. High level of omentin-1 in NVD may act as a protective factor for mother against metabolic disorders.

## Background

Omentin-1 is one of the newly diagnosed adipokines produced mainly from the adipose tissue having [1] anti-inflammatory, anti-bone loss [2] and insulin-sensitizing effect [3]. Insulin resistance, diabetes, metabolic syndrome, cardiovascular disease and inflammatory diseases are associated with low levels of omentin-1 [1]. Omentin-1 also contributes to metabolic compatibility during pregnancy [4] and its concentration is found to be higher at 11 weeks of pregnancy compared to 28th weeks of pregnancy as well as in non-pregnant women [5]. Briana et al. confirmed that the higher level of omentin-1 presence in the fetus and the newborn is attributed to the possible essential role that omentin-1 plays in promoting fetal growth and development. Omentin-1 can also contribute to energy homeostasis controlling through stimulating the use of glucose by fetus and thus promoting growth through increased insulin sensitivity [6].

In recent years, the rate of cesarean section delivery has increased [7]. Enhancement in the rate of cesarean delivery correspond to an increased rate of maternal morbidity due to bleeding, infection, hysterectomy and postpartum depression [8]. Moreover, cesarean section is associated with an increased risk of asthma, allergies, type 2 diabetes, celiac disease [9] and obesity risks during adulthood [10]. Both cesarean sections and vaginal delivery are known as an inflammatory processes which can lead to changes in the level of anti-inflammatory adipokines [11, 12].

Nowadays, the quality of life and marital satisfaction in pregnancy and postpartum period have been taken into consideration. Since postpartum period is considered a critical and transitional stage for mother and her family; physical, psychological and social adaptation are deemed essential [13]. Studies have reported that the mode of delivery has an impact on female sexuality and quality of life in the postpartum period [14, 15]. Mothers′ experience of pregnancy, delivery and postpartum period may influence their physical and psychological health; the general quality of life and their marital satisfaction [14, 16–18].

We hypothesized that omentin-1 levels, quality of life and marital satisfaction would differ with respect to modes of delivery. Considering the increasing rate of cesarean section and its complications, we decided to evaluate the maternal omentin-1 level, quality of life and marital satisfaction according to the different modes of delivery in Iranian women.

## Methods

### Design

This prospective cohort study was conducted on healthy pregnant women referring to Mardom Hospital in Tehran to have normal vaginal delivery (NVD) and cesarean section (CS) between 2017 and 2018. The sample size was calculated using the following formula and the study of Nuamah et al. [19], with 95% confidence and 80% power which equals to 41 samples in each group. Finally, with roughly 10% probability of withdrawal rate, 45 women were selected to participate in each group of the study.

$$ \mathrm{n}={\left({\mathrm{Z}}_{1-\upalpha /2}+{\mathrm{Z}}_{1-\upbeta}\right)}^2\ \left({{\mathrm{S}}_1}^2+{{\mathrm{S}}_2}^2\right):{\left({\overline{X}}_1-{\overline{X}}_2\right)}^2 $$

### Participants

**Fig. 1:**
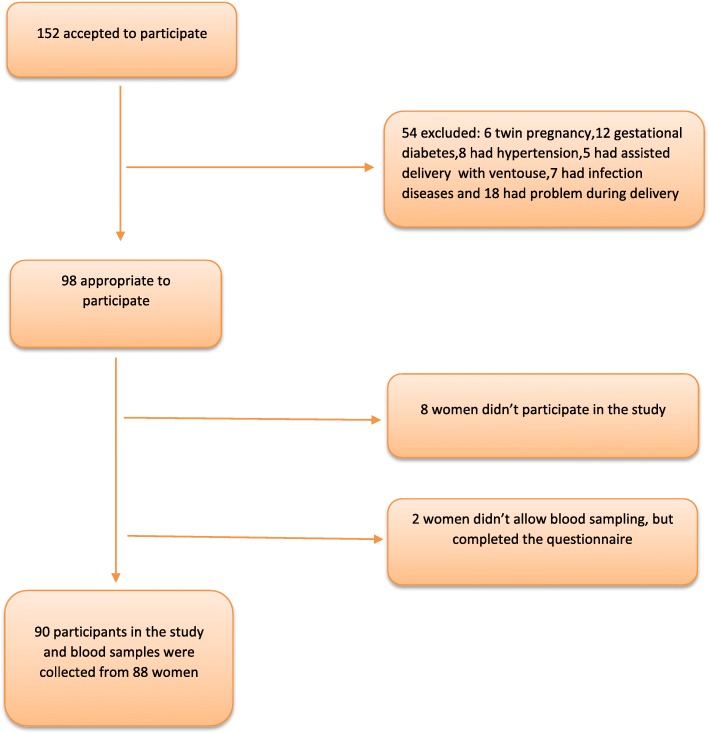
Flow-chart of the study

Ninety pregnant women were selected after examination and confirmation of the inclusion criteria and were divided into two groups of vaginal delivery (*n* = 45) versus cesarean section (*n* = 45) (Fig. 1). The gestational age in all mothers was calculated using the last menstrual period (LMP). If the date was uncertain, the first trimester ultrasound was used. Pre-pregnancy body mass index (BMI) was considered based on the BMI recorded in the first prenatal visit. The inclusion criteria comprised women aged 18–40 years, single pregnancy, gestational age of 38–42 weeks at the time of admission for delivery, having Iranian nationality; not consuming drugs, alcohol, and tobacco; having a healthy pregnancy, the absence of known pregnancy complications (e.g., preeclampsia, gestational diabetes mellitus, psychological problems and drug intake), and finally women with normal BMI (18.5–25) prior to pregnancy. The exclusion criteria included physical and psychological disorders in the mother, acute stressful events in the past 9 months (e.g., separation or loss of a family member), dystocia, fetal abnormality, infant death, preterm labor, chorioamnionitis and operative vaginal delivery. Cesarean indications comprised previous cesarean section, maternal request and breech presentation.

### Assessment of trial variables

#### Data collection was conducted using the following measures

1. Demographic questionnaire includes items on age, education, occupational status, number of children, gestational age at the time of delivery, history of pregnancy, and infant characteristics.
2. The WHOQOL-BREF questionnaire contains 24 items that are tapping into four domains. Physical health with 7 items, psychological health with 6 items, social relationships with 3 items and environmental health with 8 items. Moreover, it contains 2 items about overall quality of life and general health that are usually left unreported. The items are graded on 5- point Likert scale. The raw scores in each dimensions is converted to a 0–100 scale. Higher scores mean a better quality of life. The WHOQOL-BREF was developed by the World Health Organization (WHO) as a reduced version of the WHOQOL-100 tool [20].
3. The Kansas marital satisfaction questionnaire with 3 questions was applied to assess marital satisfaction. In this questionnaire, the total marital satisfaction score is taken into consideration; items are graded on a 7-point Likert scale and a higher score indicates a higher rate of satisfaction [21].
4. Blood samples for measuring omentin-1 were collected from the mother during the first 24 h after delivery. The samples were centrifuged (3000 rpm/5 min) and stored in − 80 °C in freezer for further analysis. Plasma omentin-1 concentrations (ng/ml) were measured by enzyme linked immunosorbent assay (ELISA) using human omentin-1 kit, ELISA, Zellbio GmbH, Ulm, Germany. The sensitivity of the omentin-1 ELISA assay was 2.5 ng/ml and the intra-assay coefficients of variation was 5.7%.

Participants completed the questionnaires in the third trimester of pregnancy and at 12 weeks postpartum period.

### Statistical analysis

Descriptive statistics were used to explore the data. We performed independent t-test and chi-square in order to conduct a between-group comparison, while for within-group comparison paired t-test was used. Pearson correlation was also applied to determine the correlation between omentin-1 levels, quality of life scores and marital satisfaction scores. The *p*-value less than 0.05 was considered significant.

## Results

Table 1 presents demographic and clinical characteristics of participants. The mean and standard deviation values of participants’ characteristics were as follows: age (27.58 ± 4.65), pre-pregnancy BMI (22.61 ± 2.14) kg/m^2^, gestational age at delivery (38.61 ± 0.72) and infant birth weight (3200.55 ± 356.91). Furthermore, 51.1% of mothers were primipara, 92.2% were housewives, 29% had academic education and none of them were reported to be hospitalized during postpartum period. Gestational age during delivery was higher among mothers with vaginal delivery than those with cesarean section.

**Table 1:** Characteristics of the women in the two groups

	Total	NVD	CS	*P*-value
**Age** (y) Mean (SD)	27.58 (4.65)	26.73 (4.81)	28.44 (4.37)	0.8*
**BMI** before pregnancy Mean (SD)	22.61(2.14)	22.7(2.19)	22.53(2.11)	0.72*
**Gestational** age at delivery Mean (SD)	38.61 (0.72)	39.17(0.61)	38.04 (0.2)	< 0.001**
**Infant birth weight** (gr) Mean (SD)	3200.55 (356.91)	3154.22(355.26)	3246.88 (356.47)	0.22*
**Education** N (%)				0.114***
academic	29 (32.2)	11 (24.4)	18 (40)	
Non-academic	61 (67.8)	34 (75.6)	27 (60)	
**Parity** N (%)				0.2***
Primipara	46 (51.1)	20 (44.4)	26 (57.8)	
Multipara	44 (48.9)	25 (55.6)	19 (42.2)	
**Employmen**t N (%)				0.23***
Yes	7 (7.8)	2 (4.4)	5 (11.1)	
No	83 (92.2)	43 (95.6)	40 (88.9)	

*Derived from two independent samples t-test

**Derived from Mann-Whitney U test

***Derived from chi-square test

**Fig. 2:**
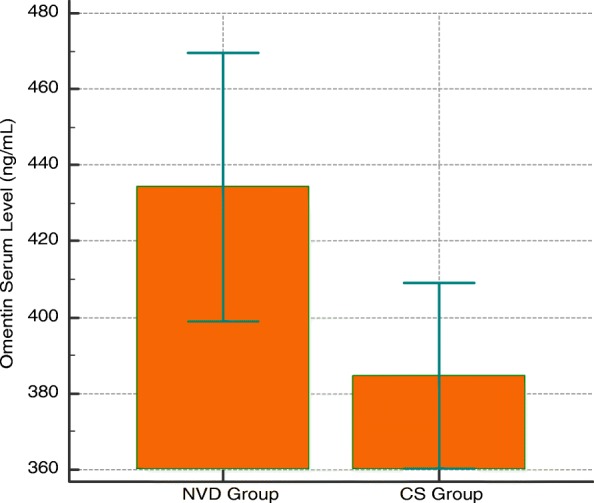
Comparison of omentin-1 levels in vaginal delivery and cesarean section groups

Maternal omentin-1 level was significantly higher in the vaginal delivery group compared to the cesarean group [mean ± SD in vaginal group 434.31 ± 116.10 ng/ml vs. 384.63 ± 80.29 ng/ml in cesarean group *p* = 0.02] (Fig. 2).

Table 2 indicates the scores in all WHOQOL-BREF subscales in two groups during the third trimester of pregnancy and 12 weeks postpartum. There was no significant difference between the two groups in terms of quality of life scores in the third trimester of pregnancy and 12 weeks postpartum period. The results of the paired sample t-test demonstrated that the score for the physical domain of QOL were significantly lower in 12 weeks postpartum compared to the third trimester of pregnancy in both groups (*p* = 0.003 in NVD group vs. *p* <  0.001 in CS group). Furthermore, the score for psychological domain of QOL in the postpartum period were significantly higher than the third trimester of pregnancy in the vaginal delivery group (*p* = 0.001).

**Table 2:** QOL between and within two groups in third trimester of pregnancy and postpartum

	Third trimester of pregnancy			Postpartum				
	CS	NVD		CS	NVD			
	Mean (SD)	Mean (SD)	P*	Mean (SD)	Mean (SD)	P*	P**	P***
Physical	66.5 (11.32)	64.44 (15.05)	0.46	60.07 (14.84)	59.28 (15.5)	0.8	< 001	0.003
Psychological	69.16 (13.48)	65.73 (16.87)	0.29	69.53 (15.4)	68.9 (16.82)	0.85	0.74	0.001
Social	72.58 (16.43)	66.29 (20.25)	0.1	70.36 (15.44)	64.81 (20.55)	0.15	0.06	0.07
Environmental	67.42 (13.3)	64.37 (14.94)	0.3	67.08 (13.21)	64.37 (14.94)	0.36	0.09	0.32

*Derived from two independent samples t-test

**Derived from paired sample t-test in CS group

***Derived from paired sample t-test in NVD group

Marital satisfaction data are demonstrated in Table 3. Marital satisfaction in the third trimester of pregnancy was significantly higher in cesarean group compared to the vaginal delivery group (*p* = 0.02). However, no significant difference was observed in marital satisfaction between the two groups at 12 weeks postpartum (*P* = 0.07). Based on the results obtained from paired sample t-test, no significant difference was observed in marital satisfaction in the third trimester of pregnancy and 12 weeks postpartum in vaginal delivery group (*P* = 0.25). Whereas, the scores recorded for marital satisfaction were significantly lower in 12 weeks postpartum compared to the third trimester of pregnancy in the cesarean group (*P* = 0.01).

**Table 3:** Marital satisfaction between and within two groups in third trimester of pregnancy and postpartum

	Cesarean	NVD	p *
	Mean (SD)	Mean (SD)	
Third trimester of pregnancy	19.28 (1.79)	17.84 (3.74)	0.02
Postpartum	18.86 (2.04)	17.73 (3.71)	0.07
P**	0.01	0.25	

*Derived from two independent samples t-test

**Derived from paired sample t-test

No significant relationship was observed between maternal omentin-1 levels and QOL in 12 weeks postpartum in both groups. Meanwhile, no significant correlation was seen between maternal omentin-1 level and marital satisfaction in 12 weeks postpartum in both groups (r = 0.04 and *p* = 0.74 in CS group vs. r = − 0.16 and *p* = 0.28 in NVD group). (Data are not shown and is only available for the first author on request).

## Discussion

The purpose of this study was to evaluate maternal omentin-1 level, quality of life and marital satisfaction in women with both vaginal delivery and cesarean section. To the best of our knowledge, this is the first study to evaluate the relationship between omentin-1 and mode of delivery. The results demonstrated that maternal omentin-1 level was significantly higher in vaginal delivery compared to cesarean section.

An acceptable explanation could be due to the uterine contraction in vaginal delivery. Omentin-1 is expressed in vascular smooth muscle cells (VSMCs) and endothelial cells (ECs) [22]. Gualillo et al. demonstrated that omentin mediates vascular health by influencing the function of endothelial cells, arterial smooth muscle cells, and macrophages in the vessel wall [23]. Furthermore, omentin prevents the adhesion of U937 monocytes to isolated vascular smooth muscle cells (VSMCs) induced by TNF-α [24].

The differences in omentin-1 level in terms of modes of delivery may also be due to ischemia of the uterine muscle wall during labor. Maruyama et al. reported that omentin stimulates the Akt-eNOS signaling pathway which in turn promotes endothelial cell function and revascularization in the ischemic conditions [25].

Based on considerable evidence of this sort, nitric oxide mediated the process of cervical ripening in labor. Omentin-1 can activate 5′-AMP-activated protein kinase and endothelial nitric oxide synthase [26].

Taken together, these findings indicate that omentin-1 may be involved in delivery process thus its concentration can also be higher in vaginal delivery.

In the present study, our findings demonstrated no significant difference in different domains of quality of life between vaginal delivery and cesarean section groups in the third trimester of pregnancy and at 12 weeks postpartum. Similarly, some studies reported no significant differences in QOL between women with vaginal delivery and cesarean section at postpartum [27, 28] . In contrast, Mousavi et al. reported that psychological and social scores of QOL were higher in the vaginal delivery than cesarean section at 8 weeks postpartum [29].

This difference in results may be attributed to differences in sample size, cultural and social differences between participants. The results of the paired sample t-test showed that the physical scores of the quality of life at 12 weeks postpartum were lower than the third trimester of pregnancy in both groups. A plausible explanation may be due to the experience of postpartum physical pain such as cesarean wound pain, perineal pain, urinary problems, hemorrhoids, movement constraints, sexual problems and sleep disorders which can reduce the score for physical domain of QOL.

In the present study, the paired sample t-test results indicated that the vaginal delivery group was reported to have a higher psychological QOL score at 12 weeks postpartum compared to the third trimester of pregnancy. Higher psychological satisfaction in vaginal delivery group may be due to pain toleration during the labor which can make the mother feel satisfied and successful.

According to the findings of the present study, no significant difference was found in marital satisfaction between vaginal delivery and cesarean section groups. Similarly, some studies demonstrated no significant association between marital satisfaction and delivery mode [30, 31]. On the contrary, Qian and colleagues reported that the incidence of sexual dissatisfaction is greater in the cesarean section compared to the vaginal delivery group [32].

A possible explanation could be the differences in sample size, methodology or demographic characteristics. According to the results of the paired sample t-test, lower marital satisfaction was observed in 12 weeks postpartum compared to the third trimester of pregnancy in cesarean section group.

Nonetheless, due to fear of sexual problems after delivery, some women are demanding elective caesarean section which increases the risk of caesarean section and lead to maternal and fetal complications. Therefore, it appears that the selection of cesarean delivery due to desirable marital satisfaction is not justified.

The results of our study showed no significant correlation between serum omentin-1, quality of life and marital satisfaction. Quality of life and marital satisfaction can be impacted by many factors including stress and energy [33, 34]. One of the roles of omentin-1 is to inhibit activation of JNK (c-Jun N-terminal kinases), hence omentin-1 can be involved in stress response, apoptosis, T-cell differentiation and expression of heat shock proteins [35, 36]. Moreover, omentin-1 can decrease mRNA expression of CART and CRH by its orexigenic effect. It is also thought that omentin is responsible for the increase of synthesis and the release of norepinephrine in the hypothalamus [37]. Yang et al. demonstrated that omentin-1 is also involved in cellular energy homoeostasis and vascular tone regulation. Omentin-1 enhances insulin-stimulated glucose uptake in human adipocytes and contributes to lipid metabolism regulation [36].

In this regard, we hypothesized that omentin-1 may be correlated with quality of life and marital satisfaction. However, our hypothesis was not confirmed in this research. Further studies are required to clarify the association between adipokines secretion, quality of life and marital satisfaction.

## Conclusion

The findings of this study revealed that maternal omentin-1 level was significantly higher in vaginal delivery than cesarean section groups. Based on the beneficial effects of omentin-1 and its inverse relationship with metabolic disorders, insulin resistance and diabetes; it may be assumed that higher levels of omentin in vaginal delivery may be useful to mother and baby. Moreover, no significant difference was found in quality of life and marital satisfaction between vaginal delivery and cesarean section groups. Given the better psychological status after vaginal delivery, it is recommended to discuss with the mothers regarding the CS without obstetric indications.

## Supplementary information

**Additional file 1.** Demographic questionnaire (PDF).

## Data Availability

Data supporting our findings can be sent upon request.

## Abbreviations

BMI: Body Mass Index
CS: Cesarean Section
LMP: Last Menstrual Period
NVD: Normal Vaginal Delivery
QOL: Quality Of Life
Rpm: Revolutions per minute
WHO: World Health Organization
