# Supplementary material for: Maternal omentin-1 level, quality of life and marital satisfaction in relation to mode of delivery: a prospective cohort study
Source: BMC Pregnancy Childbirth. 2020 Mar 2;20:136. doi: 10.1186/s12884-020-2825-2 (PMC7053092; doi:10.1186/s12884-020-2825-2)
Supplement: Supplementary file 1 — Additional file 1. Demographic questionnaire (PDF). [file 12884_2020_2825_MOESM1_ESM.pdf]

Demographic questionnaire

Name: phone number: Address:

Age:

Education: academic ◯ non-academic ◯

Employment: yes ◯ no ◯

Parity: Primipara ◯ Multipara ◯

LMP: EDC:

Gestational age at the time of delivery:

Mode of delivery: CS ◯ NVD ◯

Before pregnancy BMI:

Mother vital signs: BP: T: RR: PR:

Infant birth weight (gr):

Infant sex: boy ◯ girl ◯

Pregnancy complications: preeclampsia ◯ gestational diabetes mellitus ◯

Psychological problems ◯ drug intake ◯ alcohol or tobacco use ◯

Acute stressful event in the past 9 months ◯ dystocia ◯ Fetal abnormality ◯

Infant death ◯ preterm labor ◯ operative vaginal delivery◯ chorioamnionitis ◯

Other ◯

Cesarean indication: previous cesarean section ◯ maternal request◯ breech presentation◯
